# Randomized controlled pilot trial of supportive text messages for patients with depression

**DOI:** 10.1186/s12888-017-1448-2

**Published:** 2017-08-02

**Authors:** Vincent I. O. Agyapong, Michal Juhás, Arto Ohinmaa, Joy Omeje, Kelly Mrklas, Victoria Y. M. Suen, Serdar M. Dursun, Andrew J. Greenshaw

**Affiliations:** 1 Faculty of Health Sciences, Department of Psychiatry, University of Alberta, 1E1 Walter Mackenzie Health Sciences Centre (WMC), 8440 112 St NW, Edmonton, AB T6G 2B7 Canada; 2 Institute of Health Economics and School of Public Health, University of Alberta, Edmonton, AB Canada; 3 Department of Public Health, Alberta Health Services, Fort Mc Murray, AB Canada; 4 Research Priorities and Implementation, Research Innovation and Analytics, Alberta Health Services, Calgary, AB Canada; 5 Addiction and Mental Health Strategic Clinical Network, Alberta Health Services, Edmonton, AB Canada

**Keywords:** Depression, Mobile health, mHealth, eHealth, Supportive text messages, Randomised controlled trial

## Abstract

**Background:**

Depression is projected to be the primary cause of disability worldwide by 2030. In a recent survey, the most commonly cited unmet need among 42.4% of depressed Albertans was the lack of sufficient, accessible, and affordable counselling. Our aim was to test the efficacy of a supportive text messaging mobile health intervention in improving treatment outcomes in depressed patients.

**Methods:**

We performed a single-rater-blinded randomized trial involving 73 patients with Major Depressive Disorder. Patients in the intervention group (*n* = 35) received twice-daily supportive text messages for 3 months while those in the control group (*n* = 38) received a single text message every fortnight thanking them for participating in the study. The primary outcome of this study was: “Mean changes in the BDI scores from baseline“.

**Results:**

After adjusting for baseline BDI scores, a significant difference remained in the 3 month mean BDI scores between the intervention and control groups: (20.8 (SD = 11.7) vs. 24.9 (SD = 11.5), F (1, 60) = 4.83, *p* = 0.03, ηp2 = 0.07). The mean difference in the BDI scores change was significant with an effect size (Cohen’s d) of 0.67. Furthermore, after adjusting for baseline scores, a significant difference remained in the 3 month mean self-rated VAS scores (EQ-5D-5 L scale) between the intervention and control groups, 65.7 (SD = 15.3) vs. 57.4 (SD = 22.9), F (1, 60) =4.16, *p* = 0.05, ηp2 = 0.065. The mean difference in change mean self-rated VAS scores was also statistically significant with an effect size (Cohen’s d) of 0.51.

**Conclusions:**

Our findings suggest that supportive text messages are a potentially useful psychological intervention for depression, especially in underserved populations. Further studies are needed to explore the implications of our findings in larger clinical samples.

**Trial registration:**

ClinicalTrials.gov NCT02327858. Registered 24 December 2014.

## Background

Depression is projected to be the leading cause of disability worldwide by 2030 [1]. Chronic and recurrent nature of depression together with its traditionally labour-intensive treatment strategies make depression a difficult and expensive disorder to treat [2–7]. Enhancing treament availability and efficacy is therefore critically important from both short term and long term perspectives. This is especially relevant in low-density communities such as northern and rural Alberta where traditional therapeutic interventions might be poorly accessible. Recent evidence indicates that innovative technologies such as supportive text messaging have a large potential to be a cost effective solution to improve patient compliance, improve treatment outcomes, and provide large-scale screening and patient monitoring in a wide variety of physical and mental illnesses [8–10]. The emerging mobile health technologies could therefore help improve the current therapeutic interventions for mental ilnesses such as depression.

Mental illness and depression in particular have far reaching consequences not only to the patients and their families but also to our communities as a whole. Overall, mental illness is the most prevalent cause of disability in Canada, accountable for approximately 30% of all disability claims, 70% of total costs, and an economic loss of approximately 2.96% of GDP [1]. Several North American studies have indicated that approximately half of the mental illness short term disability claims are specifically attributable to depression [2]. Prevalence of depression among adults in Alberta is estimated as 8.4–11.9% [1, 3]. Slomp et al. (2012) identified that approximately 5.9% of Albertans sought depression-related health services directly in 2007–2008 with a total annual service cost amounting to $114.5 million or approximately $550 per patient [4]. The distribution was heavily skewed with the 1% of the most costly patients accounting for an average annual cost of $25,862 per patient [1, 4].

Less than a quarter of these depressed Albertans reported that their mental health service needs were fully met, even though two thirds of them have received a provincial mental health service in the past year [1]. In fact, recent data from the Gap Analysis of Public Mental Health and Addiction Programs (GAP-MAP) by Wild et al. indicate that, of all mental health and addiction disorders surveyed, depressed Albertans were the most likely to report unmet service needs. The most commonly cited unmet need was the lack of sufficient, accessible, and affordable counselling, which was reported by 42.4% of depressed Albertans. The GAP-MAP analysis also identified a tendency for northern Albertan residents to have higher prevalence of mental health issues [1]. Underlying reasons for these disparities are not fully understood. Nonetheless, the current evidence suggests that despite of the potentially greater mental health needs in northern Alberta, the remote northern and rural communities in Canada have to cope only with limited access to specialised mental health services [5] as compared to average Canadians.

Recent advances in telecomunications and information technology have inspired the development of a mobile health strategy (mHealth) which includes text messaging as a novel strategy to support and enhance health care and public health [6]. Since the publication of the first health text messaging study in 2002, text messaging health research has experienced rapid growth on a global scale, yet the field is still in its early phase of development and standardization [6–8]. Wireless phones are fairly ubiquitous and provide innovative opportunities for cost-effective access to large numbers of respondents with diverse demographic and social backgrounds. Mobile health technologies are particularly suitable to Alberta which has the highest wireless phone penetration in Canada at 90.1% of households (99.6% for both wireless and wireline phones) [9] and a diverse population with a significant share of non-local, transient workforce (5.7 to 6.2% of workers are estimated to be contracted from other provinces) [10] and other population cohorts who are challenging to reach and follow-up using traditional mental health treatment or research methods.

Mobile health technologies represent a convenient, expedient, and location-independent method of clinical monitoring that can be cost-effectively scaled up. These technologies also promote greater accessibility of specialized services in remote locations and offer the opportunity for enhanced non-critical care in the community. Both factors have potential for improving quality of life and treatment outcomes in patients, while containing treatment costs [11]. These properties make mobile health technologies, such as supportive text messaging, very attractive as means for alleviating unmet health service needs, including inadequate access to counselling and the improvement of treatment outcomes in patients suffering from chronic recurrent disorders, such as depression. Although evidence on the use of text messaging in depression is limited, there is a large body of literature reporting the successful use of online therapies in depression to augment cognitive behavioural therapy, monitor treatment outcomes, and improve treatment compliance (for example of a recent meta-analysis refer to Richards et al. 2012 [12]) which provides encouraging results.

In this study, we implemented an add-on supportive text messaging service for depression, a treatment intervention with demonstrated effectiveness in prior clinical trials which targeted comorbid depression and alcohol use disorder [13–16]. Our aim was to test the efficacy of using supportive text messaging as a means of improving treatment outcomes (improved mood, increased self-reported overall health, and decreased number of health service provider visits) in depressed patients.

## Methods

### Study design and participants

We conducted a single-rater blinded randomised trial of automated supportive text messages delivered through an internet application to participants’ mobile phones. The study protocol [16] was approved by the Research Ethics Board of the University of Alberta. Participants were recruited from four community mental health clinics in Fort McMurray in Alberta, Canada, between June and December 2015. Written, informed consent was obtained from each subject. The study was conducted in accordance with the Declaration of Helsinki [17] and Good Clinical Practice (WHO Guidelines) [18]. The trial is registered with clinicaltrials.gov (NCT02327858). We documented the results using the CONSORT criteria for reporting clinical trials [19].

Study participants comprised patients who satisfied the inclusion criteria, below.

1. Age 16 years and above and able to provide informed consent.
2. Were referred or re-referred from primary care to one of four community mental health clinics in Fort McMurray, Alberta, and who fulfilled the DSM-5 diagnostic criteria for Major Depressive Disorder [20] following a clinical interview by a licensed psychiatrist.
3. Patients had a mobile phone, were familiar with text messaging technology, were able to read and were available for follow-up during the study period.

Patients with comorbid alcohol use disorder and those who did not have mobile phones or refused to consent were excluded from the study.

### Patient flow

**Fig. 1:**
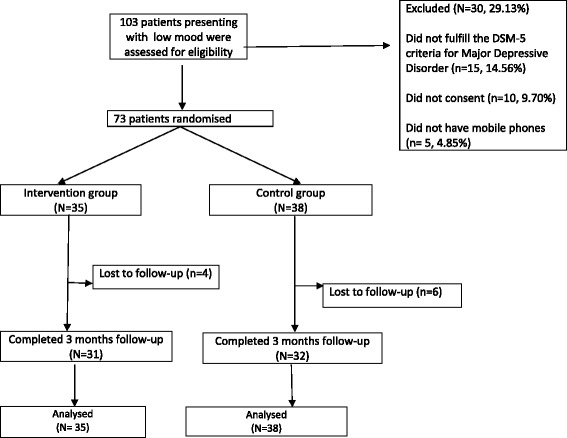
Study flow chart

One hundred and three patients were assessed for eligibility to enter the trial, of which 73 eligible patients were randomised (Fig. 1).

In all, 35 participants were allocated to the intervention group and 38 to the control group; 31 and 32 patients in the intervention and control groups, respectively, completed the study. Two patients in the intervention group and two in the control group withdrew their consent to further participate in the study before the three-month outcome assessments were due. Furthermore, two patients in the text message group and four patients in the control group could not be reached for follow-up. Those who withdrew consent for follow-up did not provide any specific explanation for their decision.

### Baseline procedures, randomisation and masking

Patients undergoing initial mental health assessments at community mental health clinics were approached by their psychiatrist to consider potential participation in the trial. Eligible consenting patients completed baseline assessments which included demographic and clinical information. Baseline depression levels were measured using the Beck’s Depression Inventory II (BDI) [21] and participants’ overall health status was assessed using the EQ-5D-5 L [22]. The EQ-5D-5 L consists of five dimensions that are mobility, self-care, usual activities, pain or discomfort, and anxiety or depression, in addition to a visual analogue scale (EQ-5D VAS) which is a vertical thermometer like scale from 0 to 100 where 0 is the worst imaginable health and 100 is the best imaginable health. All dimensions have five levels: no problems, slight problems, moderate problems, severe problems, and extreme problems/unable to do [22].

After baseline assessment, participants were randomised using a series of random numbers generated using Excel (Microsoft, Washington, USA). Participants were assigned the next available number from the randomisation sequence by a research assistant who did not participate in either the follow-up assessments or analysis. Depending on whether the randomisation number was even or odd, participants were placed in the intervention or control group, respectively. The research assistant who performed or supervised the follow-up assessments remained blind to group allocation throughout the study period.

### Interventions

Starting a day after enrolment, patients in the intervention group received twice daily unidirectional computer programmed supportive text messages for 3 months. The messages were written by cognitive behaviour therapists in partnership with patients and pre-programmed into an online software which delivered the messages at 10.00 h and 19.00 h each day. Text messages were formulated based on cognitive behaviour therapy (CBT) principles to target mood improvement. Two different messages were sent each day with no repetition of messages throughout the 90-day period. The same messages were delivered to all patients according to the day they joined the program, with no tailoring of messages to meet individual patient needs.

Examples of the text messages included the following:

- What lies behind you and what lies before you are tiny matters compared to what lies within you. Have faith in yourself and success can be yours.
- There are 2 days in the week we should not worry about, yesterday and tomorrow. That leaves today, live for today.
- Stumbling blocks can become stepping stones to a better life. You can turn adversities into opportunities. Do not be discouraged because of today’s problems.
- Letting go of resentment is a gift you give yourself, and it will ease your journey immeasurably. Make peace with everyone and happiness will be yours.
- Pay attention to activities that have a positive impact on your mood. Note these activities and refer to them when you hit a low point to improve your mood.

Patients in the control group received text messages once fortnightly thanking them for participating in the study. All study participants were encouraged to participate in the usual follow-up treatments available for patients with Major Depressive Disorder including a recommendation to self-refer for cognitive behaviour therapy either privately or with the provincial Addiction and Mental Health counselling team, attend follow-up appointments in the mental health clinics and to attend their family doctors or utilise emergency services as needed. The psychiatrists were not involved in randomising patients into either the intervention or control groups and were not involved in the follow-up assessments; procedures which were all performed by research assistants.

### Procedures at follow-up, outcome measures and hypothesis

At 3 months following their enrolment, all participants were contacted by a blinded investigator who assisted them in completing a range of assessment tools relating to the primary and secondary outcome measures listed below. More than two thirds of participants completed follow-up assessments in-person under the guidance of the research assistant after their 3 months routine follow-up psychiatric reviews in a community mental health clinic, and the remaining participants completed their assessments with the research assistant over the phone. Those completing the assessments over the phone were evenly distributed between the intervention (7) and control (8) groups, respectively. All in-person assessments involved patients self-completing outcome scales. With the over-the-phone assessments, the research assistant read out the questionnaires to the participants for them to select or indicate applicable responses.

Primary outcome measures was the mean changes in the BDI scores at 3 months from baseline. Our primary research hypothesis was that supportive text messages will reduce the three-month mean BDI scores by 30% compared to the control group. Secondary outcome measures included the mean changes in the EQ-5D-5 L instrument scores at 3 months from baseline, self-reports of the number of times participants utilised health services - including the number of visits to Family Physicians, Psychiatrists, and other specialist physicians. This also included self-reports of the number of visits to the Emergency Department, the number of CBT/counselling sessions attended, and the number of other health services utilized.

### Statistical analysis

Consistent with the idea that the study was a pilot trial, the research utilized data that could be elicited from participants who could be enrolled within the existing budget and time frame. We reached a sample size of 73 participants with Major Depressive Disorder which exceeds our target sample size of 60 reported in our published protocol for the depression trial [16].

Data were analysed on an intention-to-treat basis using SPSS version 20 for Windows [23]. Baseline demographic and clinical characteristics of the two groups were analysed using Chi-squared, Fisher’s Exact, and Student’s t-tests. Three-month BDI and EQ VAS scores were each compared between the intervention and control groups using an analysis of covariance (ANCOVA) with the treatment condition as the independent variable, baseline BDI or EQ VAS score as the covariate, and BDI or EQ VAS score at 3 months as the dependent variable, respectively. In each case, checks were conducted to ensure that there was no violation of the statistical assumptions of normality, linearity, homogeneity of variance, homogeneity of regression slopes, and reliable measurement of the covariate. For participants with missing follow-up data (four in the text message group and six in the control group), we imputed the last observation (baseline measures) [24]. We performed sensitivity analyses of covariance to explore the impact of imputation of data loss on the BDI or EQ VAS score at 3 months. We also compared categorical scores on the EQ-5D-5 L scale related to mobility, self-care, usual activities, pain/discomfort, and anxiety/depression using Chi-squared/Fisher’s Exact tests.

To explore the impact of select demographic and clinical variables on the BDI at 3 months, we performed a two-way between groups analysis of variance. Secondary outcome measures were compared using Student’s t-tests.

## Results

### Baseline demographic and clinical characteristics

Baseline demographic and clinical characteristics were similar (Table 1) in both treatment groups.

**Table 1:** Distribution of baseline demographic and clinical characteristics of participants

Variable		Intervention group N	Control group N	*P*-value
Gender	Male	10 (28.6%)	13 (34.2%)	0.60
	Female	25 (71.4%)	25 (65.8%)	
Age	≤ 25	13 (37.1%)	9 (23.7%)	0.17
	26–40	17 (48.6%)	17 (50.0%)	
	≥ 41	5 (14.3%)	12 (31.6%)	
Formal educational level	Up to high school	20 (57.1%)	19 (50.0%)	0.64
	College/University	15 (42.9%)	19 (50.0%)	
Employment status	Employed	23 (65.7%)	30 (78.9%)	0.29
	Not Employed	12 (34.6%)	8 (21.1%)	
Relationship status	In a relationship	17 (50.0%)	22 (57.9%)	0.64
	Not in a relationship	17 (50.0%)	16 (42.1%)	
On antidepressants before enrolment	Yes	19 (54.3%)	22 (57.9%)	0.82
	No	16 (45.7%)	16 (42.1%)	
On medication for chronic physical health problems	Yes	7 (20.0%)	8 (21.1%)	1.00
	No	28 (80.0%)	30 (78.9%)	
EQ-5D-5 L Mobility	No problems walking	23 (65.7%)	26 (68.4%)	0.97
	Slight problems walking	7 (20.0%)	8 (21.1%)	
	Moderate problems walking	4 (11.4%)	3 (7.9%)	
	Severe problems walking	1 (2.9%)	1 (2.6%)	
EQ-5D-5 L Self-care	No problems washing/dressing	24 (68.6%)	30 (78.8%)	0.45
	Slight problems washing/dressing	8 (22.9%)	7 (18.4%)	
	Moderate problems washing/dressing	3 (8.6%)	1 (2.6%)	
EQ-5D-5 L Unusual Activities	No problems doing usual activities	2 (5.7%)	6 (15.8%)	0.39
	Slight problems doing usual activities	12 (34.3%)	7 (18.4%)	
	Moderate problems doing usual activities	13 (37.1%)	13 (34.2%)	
	Severe problems doing usual activities	6 (17.1%)	8 21.1%)	
	Unable to do usual activities	2 (5.7%)	4 (10.5%)	
EQ-5D-5 L Pain/Discomfort	No pain or discomfort	13 (37.1%)	11 (28.9%)	0.75
	Slight pain or discomfort	12 (34.3%)	14 (36.8%)	
	Moderate pain or discomfort	6 (17.1%)	9 (23.7%)	
	Severe pain or discomfort	3 (8.6%)	4 (10.5%)	
	Extreme pain or discomfort	1 (2.9%)	0 (0.0%)	
EQ-5D-5 L Anxiety/Depression	Slightly anxious or depressed	2 (5.7%)	2 (5.3%)	0.87
	Moderately anxious or depressed	11 (31.4%)	15 (39.5%)	
	Severely anxious or depressed	15 (42.9%)	13 (34.2%)	
	Extremely anxious or depressed	7 (20.0%)	8 (21.1%)	
Mean self-rated health index (EQ VAS scores)		44.5 (19.0)	47.1 (19.1)	0.56
Mean Becks Depression Inventory-II (SD)		40.1 (8.6)	36.3 (8.8)	0.06

### Primary outcome measures

Table 2 illustrates that there was a significant relationship between the baseline BDI scores and the 3 month BDI scores (F (1, 60) = 0.12, *p* = 0.001, ηp^2^ = 0.166) with baseline BDI scores explaining about 16.6% of the variance of the 3 month BDI scores (ηp2 refers to partial eta-squared - a measure of effect size). After adjusting for these baseline BDI scores, a significant difference remained in the 3 month BDI scores between the intervention and control groups (F (1, 60) = 4.83, *p* = 0.03, ηp^2^ = 0.07). Overall, 7% of the variance in the three-month BDI was explained by the supportive text message intervention. The mean difference in the BDI scores change was also significant with a medium effect size (Cohen’s d) of 0.67.

**Table 2:** Mean scores on the primary outcome measure and one secondary outcome measure for the intervention and control groups at baseline and three-month measurement

Measure	Baseline		Three months measurement		Mean difference in change score	*P*-value (two-tailed)	Effect size (Cohen’s d)
	Intervention group	Control group	Intervention group	Control group			
Mean Becks Depression Inventory-II (SD)	40.1 (8.6)	36.3 (8.8)	20.8 (11.7)	24.9 (11.5)	−7.6 (−13.2 to −1.9)	0.01	0.67
Mean self-rated health index Using EQ VAS scores (SD)	44.5 (19.0)	47.1 (19.1)	65.7 (15.3)	57.4 (22.9)	10.7 (−0.2 to 21.5)	0.05	0.51

### Secondary outcome measures

There was a statistically significant relationship between the baseline mean self-rated VAS scores and the mean 3 month scores (F(1,60) = 11.78, *p* = 0.001) with baseline scores explaining about 16.4% of the variance in the 3 month mean scores. After adjusting for baseline scores, 6.5% of the variance (F (1, 60) = 4.16, *p* = 0.05) in the three-month mean self-rated health index was explained by the supportive text message intervention. The change in mean self-rated VAS scores was also statistically significant with a medium effect size (Cohen’s d) of 0.51.

A comparison of the five EQ-5D-5 L dimensions between the treatment groups at 3 months using the Fisher’s Exact test did not reveal statistically significant difference (*p* > 0.05 for each comparison) despite of a noticeable shift at 3 months from the EQ-5D-5 L anxiety/depression dimension where five participants in the text message group reported that they had no anxiety/depression, compared to only one participant in the control.

A subgroup analysis (Table 3) to explore interaction effects on the BDI score at 3 months did not reveal any statistically significant differences in outcomes between the different sub-groups including age, gender, educational level, employment status, and number of CBT/counselling sessions attended (no CBT/counselling session versus at least one CBT/counselling session attended and no CBT/counselling attended versus one to three counselling sessions versus more than three counselling sessions attended).

**Table 3:** Two way ANOVA comparison between patient characteristics and supportive text messaging intervention on BDI score

Independent variables		Intervention group mean BDI (SD)	Control group mean BDI (SD)	Treatment interaction F	Treatment interaction p	Subgroup main effect F	Subgroup main effect p
Gender	Male	12.0 (6.7)	25.0 (13.0)	2.44	0.12	1.23	0.27
	Female	23.4 (11.4)	24.8 (10.8)				
Age	≤ 25	22.2 (11.4)	24.8 (10.2)	0.23	0.98	0.28	0.76
	26–40	20.1 (12.2)	26.2 (11.4)				
	≥ 41	19.0 (14.0)	22.9 (13.3)				
Formal educational level	Up to high school	24.8 (12.5)	25.3 (14.0)	0.69	0.41	1.2	0.28
	College/University	15.9 (8.7)	24.6 (8.8)				
Employment status	Employed	19.6 (11.9)	25.7 (11.2)	0.27	0.60	0.001	0.98
	Not Employed	22.7 (11.4)	21.5 (13.5)				
Relationship status	In a relationship	22.3 (12.2)	23.7 (10.8)	1.10	0.30	0.11	0.70
	Not in a relationship	19.9 (11.4)	26.5 (12.6)				
On chronic physical health medication	Yes	15.0 (8.8)	23.0 (13.1)	1.06	0.30	3.80	0.06
	No	22.5 (12.0)	25.5 (11.2)				
CBT/ Counselling sessions I	Had no CBT/counselling	19.7 (9.7) (*n* = 19)	26.3 (12.7) (*n* = 14)	0.09	0.80	0.01	0.92
	Had at least one CBT/counselling session	22.5 (14.5) (*n* = 12)	24.3 (11.9) (*n* = 14)				
CBT/Counselling sessions II	Had no CBT/counselling	19.7 (9.7) (*n* = 19)	26.3 (12.7) (*n* = 14)	0.33	0.70	0.69	0.51
	Had one to three CBT/counselling sessions	27.3 (14.3) (*n* = 7)	25.9 (12.8) (*n* = 8)				
	Had four or more CBT/counselling sessions	15.8 (13.3) (*n* = 5)	22.2 (11.5) (*n* = 6)				

There were slight decreasing trends in health care utilization (mean number of physician consultations, Emergency room visits, and utilisation of counselling sessions) in the intervention group compared to the control group, however, none of them achieved statistical significance (Table 4).

**Table 4:** Health services utilisation at three months for the intervention and control groups

Variable	Intervention group	Control group	t	*P*-value
Mean number of visits to Family Physician	1.38	1.81	−0.84	0.41
Mean number of visits to Psychiatrist	1.77	2.19	−1.39	0.17
Mean number of visits to other specialists	0.35	0.55	−0.67	0.50
Mean number of visits to the Emergency Department	0.35	0.38	−0.17	0.86
Mean number of counselling sessions attended	1.51	1.54	−0.05	0.96
Mean number of other health services utilized	0.06	0.06	0.00	1.0

## Discussion

The supportive text messaging intervention in our randomised controlled study has resulted in a more than 24% additional improvement in BDI-measured mood and a more than 21% additional improvement in the aggregate self-reported health outcomes as measured on the EQ-5D-VAS score during the 3 month duration of the study. All of the participants were encouraged to follow through with their standard mental health treatment plan as prescribed by their Psychiatrists during the duration of the study. The patients were also not precluded or discouraged from seeking an appropriate treatment for physical health problems or urgent mental health issues as needed. Both of the primary outcome differences were statistically significant after correction for the heterogeneous baseline clinical profile of the two randomly assigned comparison groups.

The supportive text messaging intervention did not yield statistically significant change in the secondary outcome of health services utilization - although, on average, the intervention group sought fewer services compared to the control group. As alluded by the dimensional analysis of the EQ-5D-5 L descriptive system, a larger number of intervention patients also self-reported no anxiety/depression and less severe and extreme anxiety or depression at the 3 month follow-up. The interaction of different demographic and clinical variables on the three-month BDI score was tested in our exploratory analysis but did not yield any significant findings. Our sample was too underpowered to fully explore these trends and interactions. A larger sample size could potentially shed more colour on the health otucomes and the interplay between the health care service utilization (such as the number of counselling sessions) and the supportive text messaging intervention.

Overall, our study results indicate that supportive text messaging is a feasible and effective add-on intervention to improve treatment outcomes in depressed patients in a clinical setting in a regional health centre in northern Alberta. Supportive text messaging or similar mobile health interventions could help address the ongoing need for more assessibile mental health services for more remote or difficult to follow-up patient cohorts.

Our overall sample exhibited generally similar characteristics compared to other depression studies and clinical guidelines: the randomly selected sample of depressed patients consisted of approximately two times as many females as males and the peak demographic cohort consisted of productive-age adults [1, 25]. A notable difference compared to some other clinical studies are relatively high levels of post-secondary education (47%) and employment (73%) among our outpatients. Compared to other mobile health studies in depression such as Proudfoot et al. (2013), however, our sample had lower levels of post-secondary education (84%) and employment (84%) [26]. This is likely due to the regional economic, social, and geographic differences in the communities and patient cohorts which were sampled in the literature which might not be comparable to our sample from the oil mining dominated municipality in northern Alberta.

This is the first study of its kind in Alberta and one of a the first studies evaluating the clinical outcomes of supportive text messaging in depression rather than the indirect use of text messaging to promote treatment compliance or to monitor patients’ mood. The literature evidence on efficacy of supportive text messaging is relatively weak because few studies have directly measured mental health outcomes.

Agyapong et al. (2012) have reported successful reduction in depression scores (over 51% additional improvement compared to the control group) and a trend of prolonged cumulative abstinence duration (over 11% on average) during a 3 month supportive text messaging trial in patients recovering from comorbid alcohol use disorder and depression [13]. Twice-daily supportive messages in that trial were positively received by the patients, 83% of whom claimed that the motivational text messages have helped maintain abstinence and improve their mental well being [15]. Broom et al. (2015) have demonstrated feasability of using supportive text messages as a simple, well-accepted, and inexpensive adjunct therapy to traditional counselling in low income, minority populations with post-partum depression [27]. In this sample, 82% of mothers claimed the automated supportive text messages were relevant and 75% of them have forwarded them to others, although no direct measure of depressive symptom improvement compared to a control group was reported in that study. Whitton et al. (2015) used text messaging as part of a broader computer- and mobile-based internet mental health intervention, reporting that the most commonly used feature of the interactive internet mental health intervention was the use of short motivational messages, which was chosen by 38.4% of the patients [28]. The study did not report specifically on the efficacy of the motivational text messages on the reduction of depressive symptoms other than on the rapid and sustained overall improvement observed by the combined computer and text-messaging CBT mHealth intervention [26, 28]. Text messages tended to be more highly correlated with symptom reduction than email messages - although the difference was not statistically significant [28]. Text messages have been also used, for various purposes, in other depression studies: to cost-effectivly screen at-risk persons for depression [29], to send reminders to complete psychotherapeutic homework [30–35], and to collect data on the changes in the depressive mood or other clinical variables during treatment [28, 30, 34, 36–38].

Although literature describing treatment outcomes of supportive text messaging interventions in depression is limited, collateral evidence supports the high potential of mobile health strategies at all intervention stages of depression - from patient screening, through patient treatment and monitoring, to recurrence prevention. Current evidence suggests text messaging interventions are generally well-accepted by patients and might also be useful for underseved populations and in situations where traditional clinical interventions might not be practically feasible.

The primary limitation of this study was resource constraints which led to modest respective sample sizes (with 31 and 32 patients in the intervention and control groups, respectively, completing the three-month follow-up) as well as a lack of longer term follow-up of patients to assertain if differences observed in primary outcome measures will be sustained beyound the priod of the intervention. As a result of the relatively small sample size, our study was underpowered which has limited our ability to fully explore and interpret the findings. Nonetheless, this was one of the first studies testing the efficacy of the use of supportive text messaging to improve treatment outcomes in depressed patients. A smaller sample size is common in comparable text-messaging studies in depression, ranging between 24 and 65 patients [13–15, 27, 33]. The modest sample size of our study and the literature in general means that the results should be interpreted with caution until further replication. Another limitation was that, although the telecommunications provider’s online application has features which allowed us to track if text messages had been delivered to participants cell phones, it did not allow us to confirm if participant had actually read the text messages. Future studies should consider implementing additional mHealth techniques which would enable them to objectively track how much time participants have spent reading supportive text messages if such techniques would be ethically permissible and technically feasible in their study. This would permit researchers to investigate whether the variation in the exposure could impact the intervention outcomes in a dose-dependent way which was not possible in our study. An additional limitation is that, although not statistically significant, patients in the intervention group of our study had a higher baseline mean BID score compared to patients in the control group. This means the effects of the intervention at 3 months could in part potentially be attributed to a regression to the mean.

The major strength of this study was its robust design and conservative analysis. Our randomized controlled study followed the best clinical trial practices as outlined in Agyapong et al. (2015) [16]. We have also analyzed and reported clinical outcome measures which were collected by a study-blinded research assistant. The interactions of the different variables were tested in an exploratory analysis and the results were corrected for the inherent baseline clinical heterogeneity of our randomly assigned comparison groups. Because of the robust protocol, our study is a suitable candidate for inclusion in future meta-analyses and quantitative comparisons to future randomized clinical trials. Another strength of our study was the involvement of service users in the drafting of the supportive text messages which were used in the study.

## Conclusions

Supportive text messaging based on CBT, could potentially help alleviate the large unmet need for mental health services (such as counselling) currently reported by patients in Alberta [1] and other parts of Canada and beyound. Mobile health technologies can serve as force-multipliers for mental health providers to distribute inexpensive and more extensive mental health interventions to a wider spectrum of patients. Several studies examining text messaging have successfully demonstrated that mobile health technologies can be particularly useful for marginalized populations such as visible minorities, refugees, people of limited income, people of limited literacy, or culturally stigmatized patients [27, 29, 36, 39]. With the location-independent advantage of mobile health technologies, this could potentially make such interventions useful for some of the population cohorts in Canada who suffer from the highest prevalences of depression [39]. Implementation of text-messaging interventions has the potential for improving treatment outcomes, which in turn may decrease both the economic cost of disability and increase quality of life for patients. On a wider scale, more effective and accessible therapeutic interventions for depression could improve workplace productivity and even improve family dynamics in our communities [39]. We hope future studies will be conducted to validate our encouraging findings and explore their implications in larger clinical samples with long term follow up. Future studies may also consider including continious monitoring of psychophysiological biomarkers in order to introduce more objective measurements in outcome assessments as well as explore the dose-dependent effect of the intervention.
